# Creating the Functional Single-Ring GroEL-GroES Chaperonin Systems via Modulating GroEL-GroES Interaction

**DOI:** 10.1038/s41598-017-10499-4

**Published:** 2017-08-29

**Authors:** Melissa Illingworth, Holly Ellis, Lingling Chen

**Affiliations:** 1 Department of Molecular and Cellular Biochemistry, Indiana University, Bloomington, IN 47405 USA; 2 Biology Department, Indiana University, Bloomington, IN 47405 USA; 3 Department of Chemistry, College of Chemistry and Chemical Engineering, Xiamen University, Xiamen, 361005 China

## Abstract

Chaperonin and cochaperonin, represented by *E*. *coli* GroEL and GroES, are essential molecular chaperones for protein folding. The double-ring assembly of GroEL is required to function with GroES, and a single-ring GroEL variant GroEL^SR^ forms a stable complex with GroES, arresting the chaperoning reaction cycle. GroES I25 interacts with GroEL; however, mutations of I25 abolish GroES-GroEL interaction due to the seven-fold mutational amplification in heptameric GroES. To weaken GroEL^SR^-GroES interaction in a controlled manner, we used *groES* ^7^, a gene linking seven copies of *groES*, to incorporate I25 mutations in selected GroES modules in GroES^7^. We generated GroES^7^ variants with different numbers of GroESI25A or GroESI25D modules and different arrangements of the mutated modules, and biochemically characterized their interactions with GroEL^SR^. GroES^7^ variants with two mutated modules participated in GroEL^SR^–mediated protein folding *in vitro*. GroES^7^ variants with two or three mutated modules collaborated with GroEL^SR^ to perform chaperone function *in vivo*: three GroES^7^ variants functioned with GroEL^SR^ under both normal and heat-shock conditions. Our studies on functional single-ring bacterial chaperonin systems are informative to the single-ring human mitochondrial chaperonin mtHsp60-mtHsp10, and will provide insights into how the double-ring bacterial system has evolved to the single-ring mtHsp60-mtHsp10.

## Introduction

Molecular chaperone Hsp60 and its cochaperone Hsp10, also called chaperonin and cochaperonin, are highly conserved among the three domains of life^1^, and they are essential for cellular viability by mediating folding of cellular proteins. Hsp60 is the only molecular chaperone that is required for cell growth under normal and stressful conditions^2^. The *E*. *coli* GroEL and GroES have served as the paradigm for detailed mechanistic understandings of the chaperonin system^3–7^. GroEL consists of two heptameric rings stacked back-to-back, to form two functionally correlated folding cavities^8^. Each GroEL monomer consists of three domains. The apical domain, located at the opening of the folding cavity, binds the misfolded protein substrate and the cochaperonin GroES. The equatorial domain, located at the bottom of the folding cavity, binds ATP and forms inter- and intra-ring interactions. The intermediate domain connects the apical and equatorial domains and transmits signals between the two domains. GroES consists of one heptameric ring^9, 10^, and binds to the end of one GroEL ring to form an enclosed chamber for the folding of the substrate protein^11^. Three GroES residues I25/V26/L27 from a loop, termed the GroES mobile loop, interact with residues from the GroEL apical domain via hydrophobic interaction. The GroEL-interfacing tri-peptide sequence is highly conserved in the cochaperonin family^1^, suggesting the conserved chaperonin-cochaperonin interface. In a chaperonin-mediated folding reaction, the misfolded substrate protein is captured into the folding cavity via the apical domain. ATP binding to the substrate-loaded GroEL ring causes a series of large conformational changes, priming the ring for GroES binding, and binding of GroES sequesters the bound substrate into the newly formed enclosed folding chamber, initiating the folding process of the substrate. Binding of ATP to the GroEL ring opposite to the GroES-bound ring and the subsequent ATP hydrolysis dissociate GroES from GroEL, and release the folding substrate.

The above trans-ring allosteric effect of ATP binding/hydrolysis on GroES dissociation and substrate release is essential, and the two-ring assembly of GroEL is required for the GroEL-GroES chaperone function. Interestingly, human mitochondrial mtHsp60 exists as a single heptameric ring^12, 13^. mtHsp60 interacts with its cochaperonin mtHsp10 only transiently^14^, and as such dissociation of mtHsp10 and release of folding substrate from mtHsp60 do not require the trans-ring allostery driven by the ATP binding/hydrolysis as seen in the double ring GroEL-GroES system (above). However, the model that mtHsp60-mtHsp10 functions as a single ring^14, 15^ has been challenged. It is proposed that although mtHsp60 exists as a single heptameric ring and interacts with heptameric mtHsp10, in the course of chaperone reaction cycle, two mtHsp60-mtHsp10 complexes associate via mtHsp60 equatorial domains to form a football shape (mtHsp60-mtHsp10)_2_^16^. An mtHsp60 mutant bound with mtHsp10 was crystalized in the football conformation^17, 18^, however, the structure does not explain why the two mtHsp60-mtHsp10 molecules associate into the (mtHsp60-mtHsp10)_2_ football conformation. Thus, whether mtHsp60-mtHsp10, or broadly the chaperonin system, may operate via a single-ring mechanism is still not certain. The ability to function as single ring suggests an evolutionary adaptability of the chaperonin family.

To identify a functional single-ring chaperonin system, we set out to convert a nonfunctional single-ring GroEL variant, GroEL^SR^, by modifying its interaction with GroES. GroEL^SR^ has four mutations (R451A/E461A/S463A/V464A) to disrupt the inter-ring contact^19^. Although the GroEL^SR^-GroES cavity allows misfolded substrates to undergo folding to the native conformation^20–23^, it traps and does not release the substrates. GroEL^SR^-GroES has t_1/2_ = 300 min^−1^ ^19^, considerable longer than the ~15 s lifetime of the GroEL-GroES complex^24, 25^. Failure to release folding substrates accounts for the inability of GroEL^SR^–GroES to support cell growth^26^. Mutations in GroEL^SR^ allow the single-ring GroEL^SR^-GroES to substitute the double ring GroEL-GroES in supporting cell growth under the normal condition^27, 28^, and some mutations also support cell growth under the heat stress condition^29^. However, mechanistic understandings of these single-ring variants are limited as the mutational effects are most likely allosteric. Similarly, genetic analysis of GroES residues (G24/I25/V26/L27) on the GroEL-GroES interface has identified GroES mutants collaborate with GroEL^SR^ at lower temperatures (18 °C and 30 °C); however, little biochemical characterization of the mutational effects is available^30^.

A direct mutation on *groES* impacts all seven GroES subunits in the GroES heptamer. To avoid this inherent mutational amplification and to incorporate mutations selectively into specific GroES subunits, we generated a concatenated gene *groES* ^7^ that links seven *groES* genes to express a continuous polypeptide GroES^7^ with seven GroES modules^31^. We used *groES* ^7^ to incorporate mutations in specific GroES modules in GroES^7^ to modify the GroEL-GroES interface in a controlled manner. We hypothesized that modifying the chaperonin/cochapernonin interaction would activate the single-ring GroEL^SR^-GroES. In our earlier study, we generated GroES^7^ variants with reduced affinities for GroEL^SR^ and identified active GroES^7^ variants including GroES^7^I25D_1,4_ for GroEL^SR^-mediated folding of malate dehydrogenase (MDH). Based on these previous findings, in the current study we designed and generated comprehensive GroES^7^ variants, to systematically modulate binding of GroES^7^ to GroEL^SR^. We characterized their interaction with GroEL^SR^, their activity in assisting in protein folding and their *in vivo* chaperone function. We found that three GroES^7^ variants functioned with GroEL^SR^ in supporting cell growth under both normal and heat shock conditions.

## Results

We sought to create functional single-ring GroEL^SR^-GroES chaperonin systems that support cell growth under normal and heat shock conditions. GroEL^SR^-GroES has been shown to perform folding of substrate proteins, but its inability to release the folding substrate arrests the folding cycle, obstructing the chaperone function. To weaken GroEL^SR^-GroES interaction thereby to resume cycling of the folding reaction, here we systematically modified the GroEL^SR^-GroES interaction using a concatenated gene *groES* ^7^ we generated previously^31^.

### Mutations of GroES I25A and L27A have the same effect as I25D and L27D in abolishing GroEL-GroES interaction

**Figure 1:**
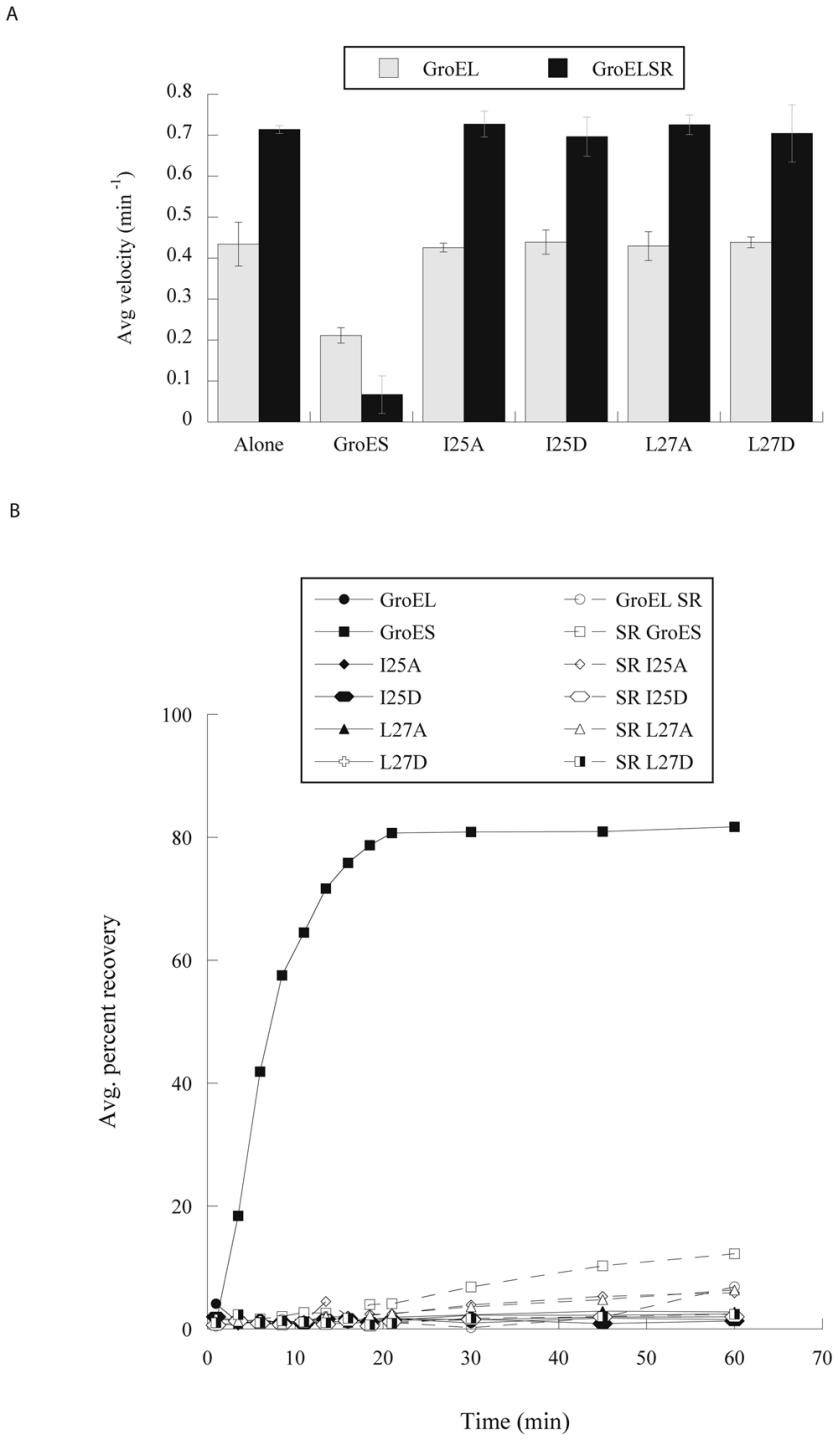
Effects of substituting I25 and L27 with Asp or Ala. (**A**) ATPase activities of GroEL (grey columns) and GroEL^SR^ (black columns) in the presence of various GroES variants. GroES inhibited the ATPase activities of GroEL and GroEL^SR^ to ~50% and ~10%, respectively. Mutations of I25A, I25D, L27A, and L27D relieved the inhibition on both GroEL and GroEL^SR^. Experiments were carried out at least three times, and error bars are standard deviations of the experiments. (**B**) Refolding of MDH in the presence of GroEL or GroEL^SR^ with various GroES variants. The enzymatic activity of native MDH is set to 100%. GroES participated in GroEL-mediated MDH folding with ~80% yield, and in GroEL^SR^-mediated MDH folding with ~10% yield. The GroES variants are associated with minimal MDH folding yield.

The GroEL-GroES interaction can be characterized via three assays: the ATPase activity, since binding of GroES inhibits GroEL’s ATPase activity by 50%^25, 32, 33^, the enzymatic activity of malate dehydrogenase (MDH) since efficient folding of MDH requires not only the formation but also dissociation of the GroEL-GroES folding cavity^34^, and measurement of dissociation constant (K_d_). GroES interacts with GroEL via a tri-peptide I25/V26/L27 region^11^, and our previous study showed mutations of either I25D or L27D but not V26D in GroES abolish GroES’s interaction with GroEL^31^. Specifically, we showed that both GroESI25D and GroESL27D mutants did not inhibit GroEL’s ATPase activity, did not participate in GroEL-mediated MDH folding, and no stable GroEL-GroES complex could be isolated. We reasoned that a conserved mutation to Ala would have a less detrimental effect and would not completely abolish the hydrophobic GroEL-GroES interaction. As shown in Fig. 1A, both GroESI25A and GroESL27A did not inhibit GroEL’s ATPase activity, suggesting that both Ala mutations abolished GroEL-GroES interaction. Additionally, no MDH activity was observed in either GroEL-GroESI25A or GroEL-GroESL27A (Fig. 1B), indicating that neither GroESI25A nor GroESL27A collaborated with GroEL in assisting folding of MDH. Finally, we measured GroEL-GroES interaction using microscale thermophoresis (MST). Both I25A and L27A mutations decreased GroES’s binding affinity to GroEL by >1,000 fold from Kd’s values of 3.83 (±0.93) nM to >5 uM (Supplementary Table S1).

We next evaluated the Ala mutational effect on GroES’s interaction with the single-ring GroEL^SR^. Wild type GroES has a strong binding affinity for GroEL^SR^ as shown in the three aspects: it inhibits the ATPase activity of GroEL^SR^ by ~90%^19^, GroEL^SR^-GroES traps the refolding MDH resulting in lack of MDH activity^21^, and the GroEL^SR^-GroES complex is highly stable with a slow dissociation rate^19^. We have shown that either I25D or L27D mutations in GroES abolish GroEL^SR^–GroES interaction^31^. Figure 1A shows that like GroESI25D and GroESL27D, neither GroESI25A nor GroESL27A affected ATP hydrolysis of GroEL^SR^, suggesting that they did not interact with GroEL^SR^. Also similar to their Asp counterparts, neither GroESI25A nor GroESL27A collaborated with in GroEL^SR^ in actively refolding MDH (Fig. 1B). Finally, like the Asp variants, the GroES Ala variants did not show binding affinity for GroEL^SR^ based on MST (data not shown).

Together, the Ala mutations at I25 and L27 drastically abolished GroES’s binding to both GroEL and GroEL^SR^, the same effect as observed with the Asp mutations. These findings suggest that the hydrophobic residue with extended side chain at positions 25 and 27 are important for productive GroES-GroEL interaction. Consistent with this finding, residues at these two positions in the GroES sequences from bacteria to human are mostly Ile and Leu and sometimes Met^1^.

### One-, two- and three I25A or I25D mutated GroES modules in GroES^7^ gradually decreased GroEL-GroES^7^ interaction

The drastic mutational effect on abolishing GroEL-GroES interaction can be explained by the amplification effect that one mutation in *groES* affects all seven subunits in GroES. To control GroES’s affinity for GroEL in a systematic manner, we created a gene *groES* ^7^ in our previous study^31^. *groES* ^7^ links seven copies of *groES* to express a continuous polypeptide GroES^7^ with seven GroES modules, allowing us to mutate specific residue(s) at desired GroES module(s) in GroES^7^ to create combinations of the mutated and wild type GroES modules. We have shown that mutations of either I25D or L27D in one (1^st^), two (1^st^ and 4^th^) and three (1^st^, 4^th^ and 7^th^) GroES modules in GroES^7^ gradually decrease GroEL-GroES^7^ interaction and steadily relieve the strong inhibition on ATPase activity of GroEL^SR^. Since I25D mutation displays greater mutational effect than L27D mutation^31^, we focused the current study on investigating the I25 mutational effects in GroES^7^. We generated extensive GroES^7^ variants with two- or three-I25D or I25A GroES modules. There are three unique ways to place two mutated GroES modules, so we had all six two-mutated variants, GroESI25A_1,2_, GroESI25A_1,3_, GroESI25A_1,4_, GroESI25D_1,2_, GroESI25D_1,3_ and GroESI25D_1,4_. We generated four variants with three mutated GroES modules: GroESI25A_1,4,6_, GroESI25A_1,4,7_, GroESI25D_1,4,6_ and GroESI25D_1,4,7_.

**Figure 2:**
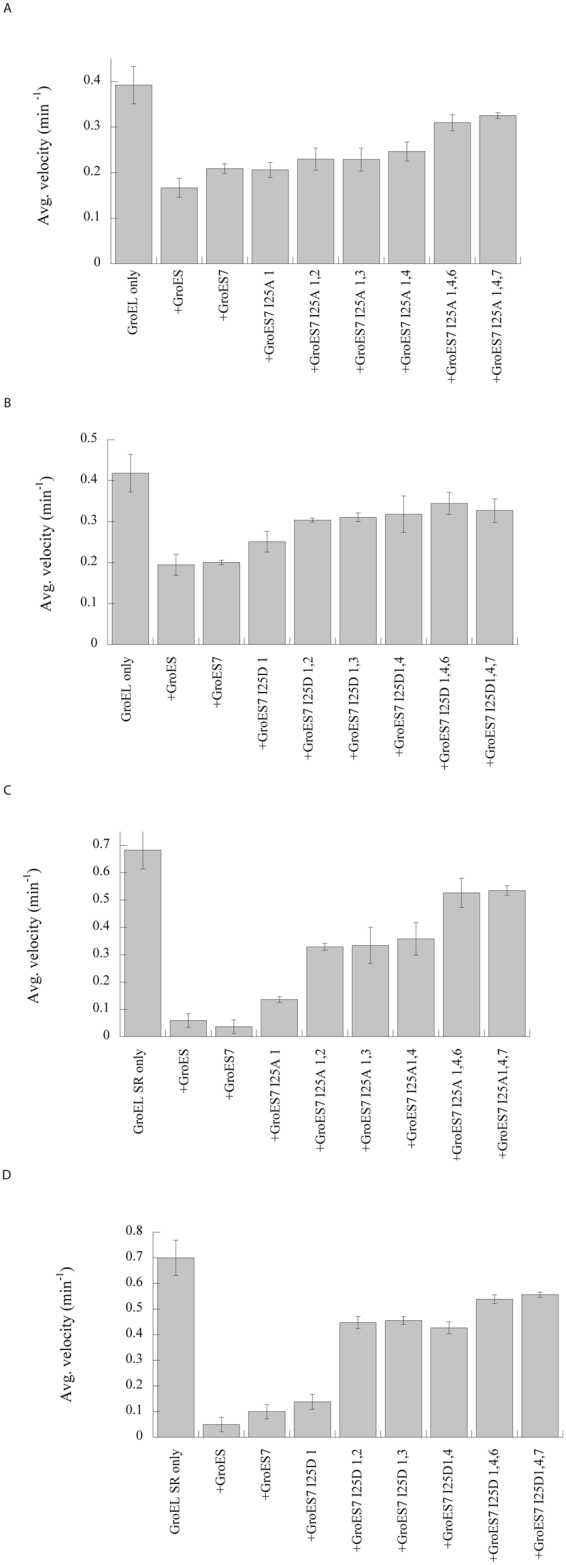
Effects of substitutions in GroES^7^ on the ATPase activities of GroEL (**A** and **B**) and GroEL^SR^ (**C** and **D**). Experiments were repeated more than three times, and error bars are the standard deviations among the different measurements. The data are summarized in Supplementary Tables S1 and S2.

**Figure 3:**
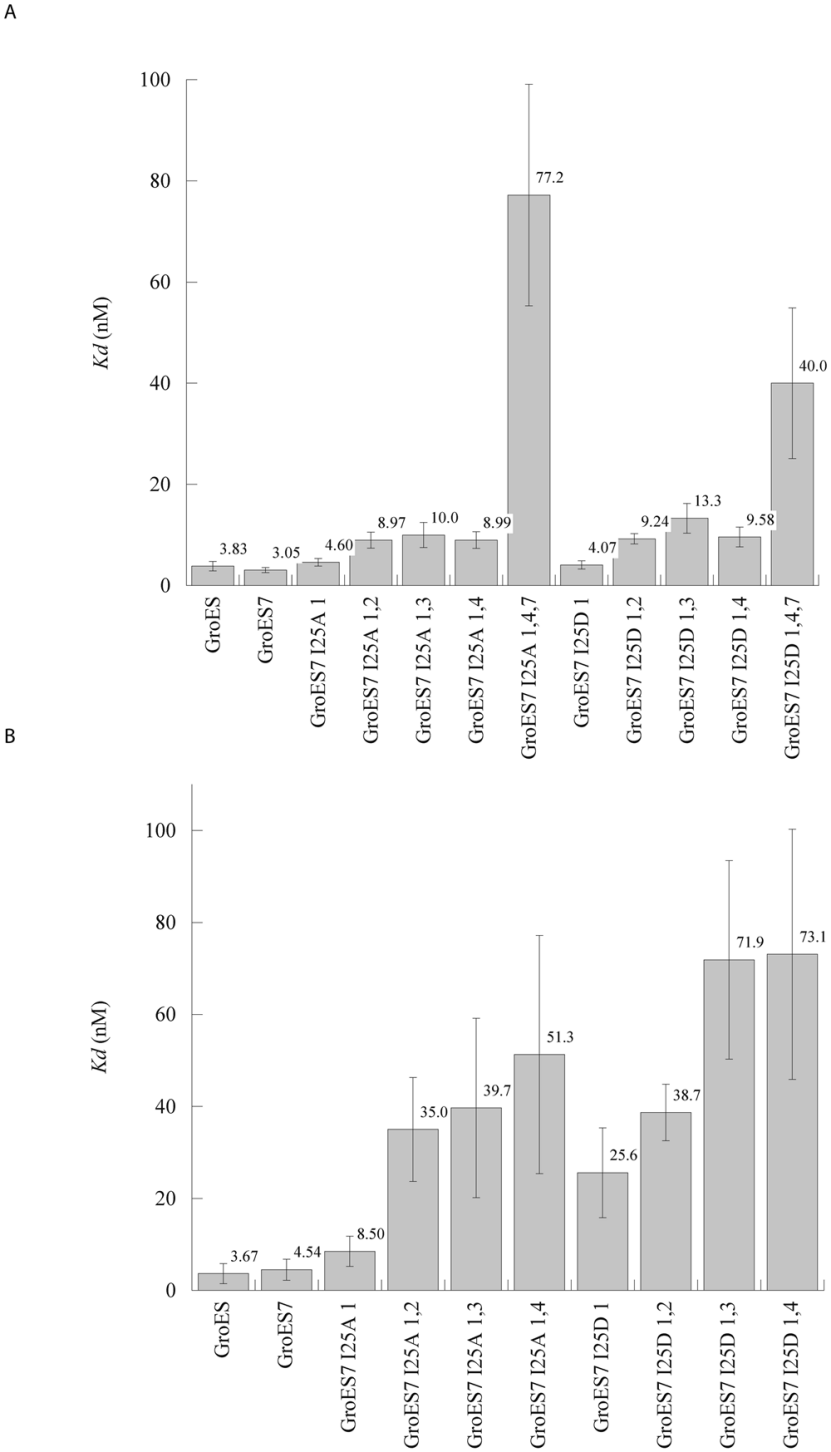
Binding of GroES, GroES^7^ and GroES^7^ variants to GroEL (**A**) and GroEL^SR^ (**B**) via MST experiments. Experiments were repeated more than three times, and error bars are the standard deviations among the different measurements. The data are summarized in Supplementary Tables S1 and S2.

As the number of either the I25A or I25D modules increased in GroES^7^, the GroEL-GroES^7^ interactions decreased. For the I25A series, one mutated module, GroESI25A_1_, inhibited ATPase of GroEL to a level (53.2%) higher than that of GroES (42.5%). Two-mutated modules, GroESI25A_1,2_, GroESI25A_1,3_ and GroESI25A_1,4_, had markedly reduced inhibitions with the remaining ATPase activities of 58.5–62.8%. Three-mutated modules, GroESI25A_1,4_,_6_ and GroESI25A_1,4_,_7_, further relived the inhibition with the remaining ATPase activity of 79.0–82.9% (Fig. 2A and Supplementary Table S1). As expected, binding affinity of GroES^7^ for GroEL decreased as the number of the mutated module increased, with one-mutated module only moderately affecting affinity, two-mutated modules reducing the affinity by two folds, and three-mutated modules by more than 25 folds (Fig. 3A and Supplementary Table S1).

**Figure 4:**
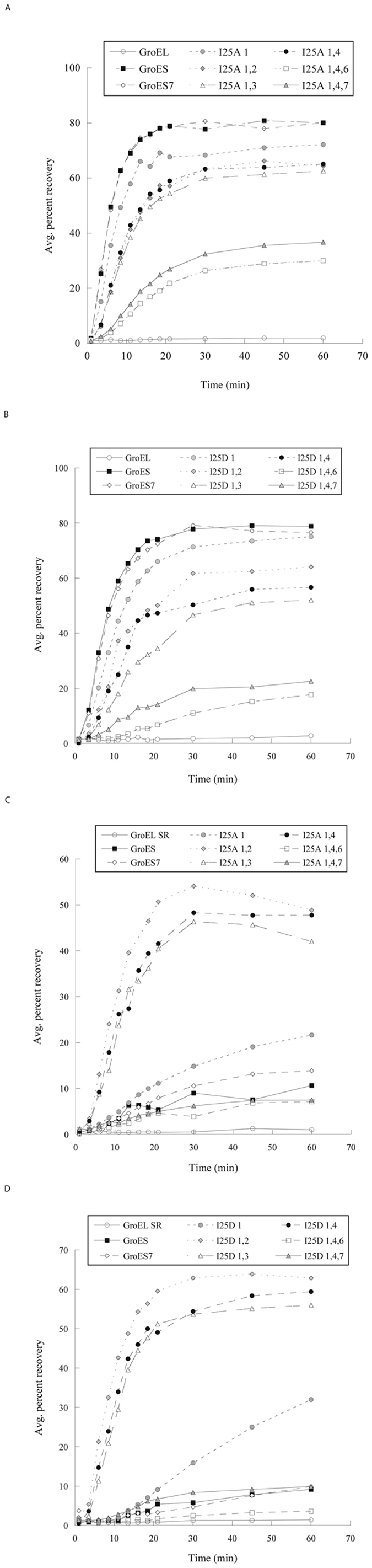
Effects of substitutions in GroES^7^ on the GroEL-mediated (**A** and **B**) and GroEL^SR^-mediated MDH folding (**C** and **D**). The enzymatic activity of native MDH is set to 100%. Experiments were repeated more than three times, and representative data from individual runs were shown. The MDH yields are summarized in Supplementary Tables S1 and S2.

An increased number of the mutated GroES module also decreased both the yield and kinetics of GroEL-mediated MDH folding (Fig. 4A). One mutated module, GroESI25A_1_, decreased the folding yield from 80% to 72% and the folding kinetics modestly. Two mutated modules, GroESI25A_1,2_, GroESI25A_1,3_ and GroESI25A_1,4_, reduced the folding yield further to 62–65%, and slowed the folding kinetics by ~50% (Fig. 4A). Three mutated modules, GroESI25A_1,4,6_ and GroESI25A_1,4,7_, decreased the yield drastically to 30–37% and reduced the folding kinetics by ~85%. Paralleled trends in gradual increase in ATPase activity (Fig. 2B), decrease in binding affinity (Fig. 3A) and decrease in MDH folding activity (Fig. 4B) were found in presence of GroES^7^ variants with one-, two- and three-mutated GroESI25D modules (Supplementary Table S1).

### Large mutational effect of GroES^7^ on GroEL^SR^-GroES^7^ interaction

The above mutational effects of GroES^7^ were more pronounced in interactions with the single-ring GroEL^SR^ than with the double-ring GroEL. For the I25A mutation series, one mutated module, GroESI25A_1_, lifted the inhibition on ATPase of GroEL^SR^ from ~90% to 80% (Fig. 2C and Supplementary Table S1). Two mutated modules, GroESI25A_1,2_, GroESI25A_1,3_ and GroESI25A_1,4_, drastically relieved the inhibition to ~50%, a level as seen in the canonical GroEL-GroES system where GroES inhibits the ATPase activity of GroEL by 50%^19, 24, 25^. Three mutated modules, GroESI25A_1,4,6_ and GroESI25A_1,4,7_, further relived the inhibition to ~20%. In line with these ATPase studies of GroEL^SR^, large mutational effects of GroES^7^ on the binding affinity for GroEL^SR^ were observed. One mutated module, GroESI25A_1_, reduced binding affinity for GroEL^SR^ by ~50% (K_d_ of 8.5 ± 3.3 nM from ~3.7 ± 2.2 nM), which is comparable to the effect of the two-mutated modules on binding affinity for the double ring GroEL (Fig. 3 and Supplementary Tables S1 and S2). Two mutated modules, GroESI25A_1,2_, GroESI25A_1,3_ and GroESI25A_1,4_, markedly reduced the binding affinity of GroES^7^ for GroEL^SR^ by > 10 folds (Fig. 3b and Supplementary Table S2), which is comparable to the effect of the three-mutated module of GroESI25A_1,4,7_ on binding affinity of GroES^7^ for GroEL. Three mutated modules, GroESI25A_1,4_,_6_ and GroESI25A_1,4,7_, appeared to abolish the binding affinity for GroEL^SR^ as no detectable binding was observed.

### Mutational effects on GroEL^SR^-mediated MDH folding

One mutated module in GroES^7^, GroESI25A_1_ and GroESI25D_1_, increased the GroEL^SR^-mediated MDH folding yield from <10% to 20–30% (Fig. 4C and D; Supplementary Table S2). Two mutated modules, GroESI25A_1,2_, GroESI25A_1,3_ and GroESI25A_1,4_, GroESI25D_1,2_, GroESI25D_1,3_ and GroESI25D_1,4_, further improved the MDH folding with both the yield and kinetics comparable to the canonical double ring GroEL-GroES (Fig. 4C and D; Supplementary Tables S1 and S2). However, adding a third mutated module in GroES^7^I25A_1,4_ and GroES^7^I25D_1,4_, to create GroES^7^I25A_1,4,7_, GroES^7^I25A_1,4,6_, GroES^7^I25D_1,4,7_, and GroES^7^I25D_1,4,6_, reverted the folding yield to the minimum as seen with GroES (Fig. 4C and D). These findings using different types of mutation, Ala and Asp mutations, and extensive combinations of the mutated module recapitulate our previous results using a representative group of GroES^7^ variants, GroES^7^I25D_1_, GroES^7^I25D_1,4_ and GroES^7^I25D_1,4,7_^31^. Thus, we concluded that GroES^7^ variants with two-mutated modules, irrespective to the positions of the mutated modules and the types of mutation, were effective and efficient in GroEL^SR^-mediated MDH folding.

### Mutational effects on *in vivo* chaperone function

**Figure 5:**
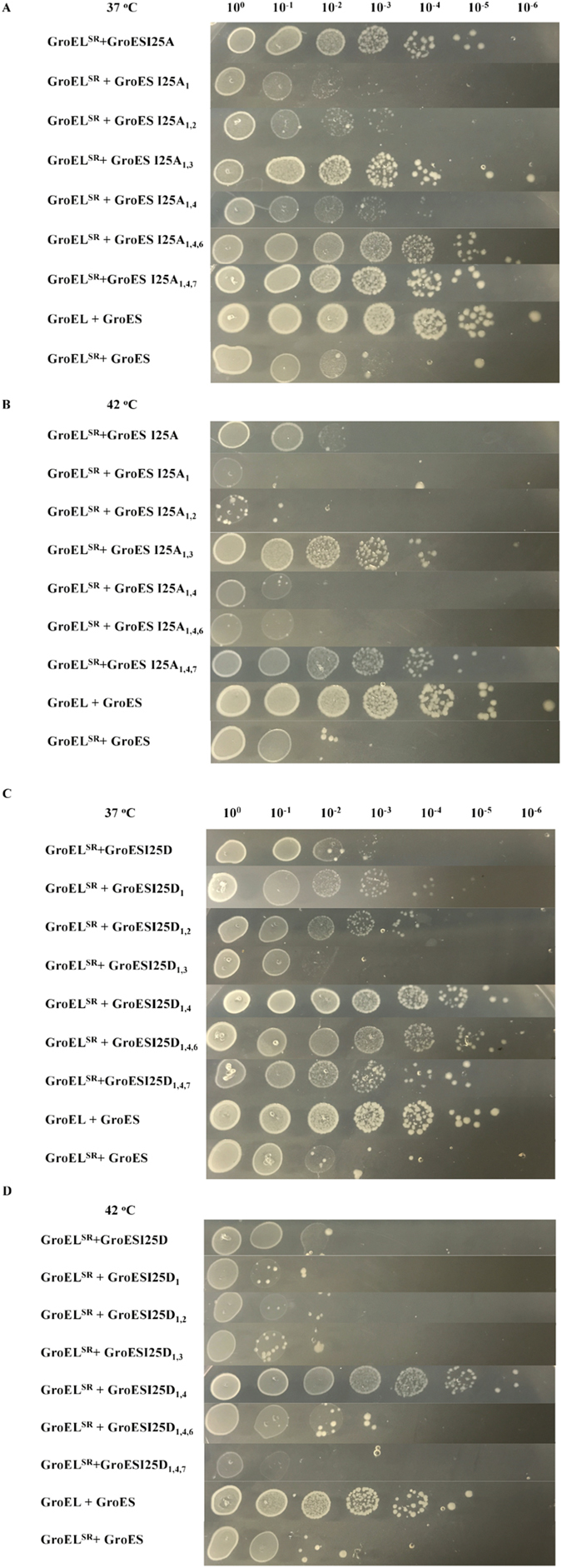
*In vivo* chaperone function of GroEL^SR^-GroES^7^ variants at 37 °C (**A** and **C**) and 42 °C (**B** and **D**) via assaying growth of *E*. *coli* MGM100 cells.

We reasoned that the MDH-folding active chaperonin systems should have chaperone function, and examined whether the single-ring GroEL^SR^-GroES^7^ systems were able to substitute the canonical double ring GroEL-GroES in supporting growth via a conditional lethal *E*. *coli* strain MGM100^35^. Interestingly, the ability to refold MDH is not correlated with the *in vivo* chaperone function. For example, of the six GroES^7^ variants with two-mutated modules, only GroES^7^I25A_1,3_ and GroES^7^I25D_1,4_ were able to function with GroEL^SR^ at both the optimal temperature of 37 °C and under heat shock temperature of 42 °C (Fig. 5). The three GroES^7^ variants with two-mutated modules, GroES^7^I25A_1,2_, GroES^7^I25A_1,4_ and GroES^7^I25D_1,2_, might partially function with GroEL^SR^ at 37 °C, but they did not function with GroEL^SR^ under heat shock. One GroES^7^ variants with two-mutated modules, GroES^7^I25D_1,3_, did not function even at 37 °C. In addition, all four GroES^7^ with three-mutated modules, despite their little activity in MDH folding, functioned with GroEL^SR^ at 37 °C; moreover, one of them, GroES^7^I25A_1,4,7_-GroEL^SR^, was functional also at 42 °C. Finally, GroESI25A, with all seven mutated subunits, was functional with GroEL^SR^ at 37 °C, despite its inability to interact with GroEL^SR^ based on ATPase and MST assays and to refold MDH. The reason for the lack of correlation between MDH folding activity and *in vivo* chaperone function is not clear, however, it is noted that MDH is not the authentic cellular substrate for GroEL-GroES although MDH folding assay is commonly used in the chaperone field. Nevertheless, we identified three GroES^7^ variants, GroES^7^I25A_1,3_, GroES^7^I25D_1,4_ and GroES^7^I25A_1,4,7_, to function with the single-ring GroEL^SR^ in supporting cell growth under both the optimal and heat shock conditions. These GroES^7^ variants have mutations on the interface with GroEL that directly weaken the GroEL^SR^-GroES interaction, providing the molecular basis for functional single-ring chaperonin system.

## Discussion

The chaperonin system is essential for cellular viability by mediating folding of cellular proteins. The double-ring assembly of bacterial GroEL is required for the chaperone function, because the trans-ring allostery is required to dissociate the stably formed GroEL-GroES complex and to release the enclosed folding substrate protein. The human mitochondrial mtHsp60 may adopt a distinct single-ring mechanism because mtHsp60 exists as a single heptameric ring and has a lower affinity for mtHsp10. A recent model for mtHsp60-mtHsp10 suggests, however, that during the mtHsp60-mtHsp10 reaction cycle two mtHsp60-mtHsp10 complexes associate to form a football shape (mtHsp60-mtHsp10)_2_, suggesting that mtHsp60-mtHsp10 may not truly function in a single-ring mechanism. We sought to show that the chaperonin system may rely solely on the single-ring mechanism to execute the chaperone function, by activating a single-ring form of GroEL, GroEL^SR^.

GroEL^SR^ is not functional with GroES because without the allostery from the absent second ring the tight GroEL^SR^–GroES interaction traps folding protein substrates and arrests the chaperone reaction cycle. To obtain functional single-ring GroEL^SR^-GroES by selectively weakening GroEL^SR^–GroES interaction in a systematic manner, we utilized a novel reagent *groES* ^7^, that links seven *groES* to express GroES^7^ with seven genetically independent GroES modules. We created extensive GroES^7^ variants with one, two and three modules of either GroESI25A or GroESI25D mutations. We systematically characterized mutational effect on various activities of GroEL and GroEL^SR^. We found that as the number of the mutated modules increased the inhibition on ATPase activity, the binding affinity and MDH folding activity of GroEL steadily decreased, suggesting that gradual decrease in GroEL-GroES^7^ interaction. Decreases in inhibiting ATPase activity of and in binding affinity for GroEL^SR^ were greater than as seen in GroEL, and suggested that GroES^7^ variants with mutated modules resumed a recyclable reaction with the single ring GroEL^SR^. Notably in mediating MDH folding, GroES^7^ variants with two mutated modules were active with GroEL^SR^ with both the folding yield and kinetics comparable to the canonical double ring GroEL-GroES. Importantly, we found three GroES^7^ variants, GroES^7^I25A_1,3_, GroES^7^I25D_1,4_ and GroES^7^I25A_1,4,7_, were functional with GroEL^SR^ under both normal and heat shock temperatures.

The chaperonin-cochaperonin interaction is central for chaperonin to function as single ring. Early genetic screens isolated GroEL^SR^ variants that are functional with GroES at 37 °C^27^, and the chaperonin-cochaperonin interaction in these functional GroEL^SR^-GroES systems is much weaker compared to GroEL-GroES^27, 29, 36^. Since these mutated GroEL^SR^ residues are not located in the GroEL-GroES interface, the mutational effects on GroEL^SR^-GroES interaction are presumably allosteric and molecular basis for the allosteric effect remains unclear. Direct mutations on the GroEL^SR^-GroES interface, G24/I25/V26/L27, in the GroES mobile loop, identified GroES variants GroESI25F and GroESI25L that appear functional with GroEL^SR^ at 37 °C^30^. Both variants decreased inhibition on the ATPase activity of GroEL^SR^, suggesting their reduced interaction with GroEL^SR^; however, no further characterizations on the GroEL^SR^-GroES interaction have been reported. Our abilities to directly modulate the chaperonin-cochaperonin interface, shown in this and previous^31^ studies, confirm that reduced chaperonin-cochaperonin interaction is key to create functional single ring. We found that modifying two or three of the seven individual GroEL-GroES interactive surfaces is effective in rendering single ring GroEL^SR^-GroES functional *in vivo*. Positions of the modified individual interfaces, 1,2, 1,3 and 1,4 or 1,4,6 and 1,4,7, have different effects on functionality of GroEL^SR^-GroES. These findings support the structural observations that each of the GroEL-GroES interfaces, including conformations of both the GroES mobile loop and the GroEL Helix H and I, is unique^37^. In terms of interaction strength, we found that the working chaperonin-cochaperonin interaction for a functional single ring GroEL-GroES-based system follows the Goldilocks principle: interaction must not be too loose or too tight. Our studies provide the first step for future mechanistic investigations on the Goldilocks chaperonin-cochaperonin interaction of the single-ring chaperonin system.

Our results that the chaperonin system may rely on the single-ring mechanism are informative to the human mitochondrial chaperonin mtHsp60-mtHsp10. mtHsp60 exists predominately as single heptameric ring^12^ in equilibrium with the monomeric form^16^. The lack of the double ring conformation is consistent with its absence of the two conserved salt bridges (K105-D435 and E461-R452; residue naming according to GroEL) that are important to stabilize the inter-ring interaction^38^. In addition, compared to the stable GroEL-GroES complex (K_d_ of 0.1–26 nM^20, 33, 34, 39^, or 3.83 ± 0.93 nM of this study, in the presence of ADP), the reduced mtHsp60-mtHsp10 interaction^14^ supports the dispensable role of a second ring in the chaperoning reaction cycle. Further support for mtHsp60-mtHsp10 functioning in a single ring mechanism comes from the functional single ring GroEL^SR^/mtHsp60 chimera^14, 15^. Interestingly, in the presence of both ATP and mtHsp10 two mtHsp60 heptameric rings appear to associate, forming the football (mtHsp60-mtHsp10)_2_ conformation^16^. Investigations on whether mtHsp60 undergoes an association to form a double ring conformation in the mtHsp60-mtHsp10 reaction cycle are hindered by the dynamic nature of mtHsp60 quaternary assembly and mtHsp60-mtHsp10 interaction. Genetic screens identified a mutant mtHsp60^E321K^ with high affinity for mtHsp10, forming stable mtHsp60^E321K^-mtHsp10 and arresting the chaperone cycle^40^, reminiscent of GroEL^SR^ arresting GroEL-GroES cycle. mtHsp60^E321K^-mtHsp10 crystalized in the football conformation^17, 18^, that is, two heptameric mtHsp60^E321K^-mtHsp10 complexes associate via mtHsp60^E321K^. The two mtHsp60^E321K^ heptameric rings interface via the equatorial domains as seen in GroEL, and as expected no charge-charge interactions in the place of the two conserved inter-ring salt bridges (K105-D435 and E461-R452) are observed. Strikingly, the inter-ring interface in mtHsp60^E321K^ is twice as that in the naturally occurring double-ring GroEL. Such extensive inter-ring interface suggests a stable, GroEL-like double ring conformation, which is in direct contrast to the observed, single-ring conformation. Such extensive inter-ring interface may suggest cross-ring communication and regulation, justifying the assembly of the double ring conformation for biochemical activities. For example, the ATP-induced cross-ring allostery manifests in various aspects in GroEL-GroES. Notably, binding of ATP to one GroEL ring prevents ATP binding to the opposite ring^41^, and ATP binding in one ring initiates GroES dissociation from the opposite GroEL ring^21^. For mtHsp60-mtHsp10, the negative ATP binding cooperativity has not been reported, and mtHsp10 dissociates readily from mtHsp60 due to the weak interaction. Besides the lack of biochemical support, structure of (mtHsp60^E321K^-mtHsp10)_2_ does not offer structural insights into either cross-ring communication or the double ring assembly of the football conformation important for the mtHsp60-mtHsp10 reaction cycle. Thus, the mechanistic significance for association of two mtHsp60-mtHsp10 to form a football conformation of (mtHsp60-mtHsp10)_2_ is not clear, and whether the football conformation is the productive intermediate in the chaperone cycle is unknown. However, considering the complex cellular conditions, it is probable that two heptameric mtHsp60-mtHsp10 (mtHsp60) molecules might associate to form the double ring assembly as seen in structure of (mtHsp60^E321K^-mtHsp10)_2_. The cellular conditions favorable for molecular association include the abundance of cellular chaperonin (2.6 μM for GorEL^42^), the high concentration of cellular macromolecules (300–400 mg/ml in *E*. *coli* ^43^) and the macromolecular crowding effect^43^ that results in increasing the effective concentration of mtHsp60. While investigations on these important mechanistic aspects of mtHsp60-mtHsp10 continue, here we, in conjunction with previous studies^14, 15, 27–29^, show that the chapreonin can rely on the single-ring mechanism to function. Our results demonstrate the mechanistic adaptability of the chaperonin system, and our functional single ring GroEL^SR^-GroES^7^ variants will provide valuable tools to study the molecular evolution of this ancient protein family from bacterial double-ring to human mitochondrial single-ring conformations.

## Methods

### Protein expression and purification

*groEL* and *groEL* ^*SR*^ (GroEL R452A/E461A/S463A/V464A) were in pTrc vector, *groES* was in pET3b, and *groES* ^7^ and the *groES* ^7^ variants were in a modified pET28b^31^. *E*. *coli* BL21(DE3) cells were used to express the proteins. Conditions for cell growth, induction of protein expression, and protein purification are described in ref. 31. To remove the residual proteins bound to GroEL or GroEL^SR^, the chaperonins (1 mg/ml) were dialyzed against 50 mM TrisCl pH 7.5, 1 mM EDTA and 30% methanol, loaded onto a FastQ column (GE Healthcare), and eluted with 0–1 M NaCl gradient. The chaperonin-containing fractions were combined, dialyzed with TEA buffer (50 mM triethanolamine 7.5, 50 mM KCl and 20 mM MgCl_2_) and 0.1% NaN_3_ at 4 °C overnight. The purified chaperonins were verified with minimal Trp fluorescence.

### ATPase activity assays via Malachite green

Chaperonins and cochaperonins were dialyzed into TEA reaction buffer containing 50 mM KCl and 20 mM MgCl_2_, to 0.125 μM tetradecameric chaperonins, and 0.3 μM heptameric cochaperonins. ATPase activity was measured via malachite green as described in ref. 31 at room temperature (22 °C) with 2 mM ATP as the starting concentration. Absorption at 660 nm (A_660_) was measured, and the final A_660_ values were averaged over three readings. The amount of hydrolyzed free phosphate was derived from a standard curve, and the hydrolysis rate was normalized to GroEL monomer. At least three independent experiments were performed.

### MDH refolding assay

Chaperonins and cochaperonins were dialyzed into TEA reaction buffer. Malate dehydrogenase (Roche) was unfolded in TEA buffer including 3 M GdmHCl to a final concentration of 36.7 μM (monomeric MDH) for 60 minutes prior to the experiments. MDH refolding assay via monitoring the enzymatic activity of the refolded MDH at A_340_, was described in ref. 31. The final protein concentrations were 1 μM of GroEL or 2 μM GroEL^SR^, 4 μM of cochaperonin, and 0.7 μM of monomeric MDH. The enzymatic activity of native MDH was set to 100%, and at least three independent experiments were performed.

### Chaperonin-cochaperonin binding via microscale thermophoresis (MST) assay

GroES, GroES^7^ and GroES^7^ variants were fluorescently labeled with DyLight^TM^ 650 NHS Ester Amine Reactive Dye (ThermoScientific) according to manufacturer’s protocol. The labeled chaperonin was separated from the free dye using MidiTrap (GE Healthcare) followed by dialysis (to 50 mM TrisCl pH 7.5, 100 mM KCl, 10 mM MgCl_2_, and 1 mM EDTA), and its concentration was measured using the Bradford assay. For each unlabeled proteins (GroEL or GroEL^SR^), a serial dilution of 15 samples were prepared in the binding buffer (50 mM TrisCl pH 7.5, 100 mM KCl, 10 mM MgCl_2_, 1 mM EDTA, 2 mM ADP, and 0.5 mg/mL BSA). 10 ul of the unlabeled protein was incubated with 10 ul of the labeled cochaperonin for 30 min, and the solution was loaded into a glass capillary (NanoTemper Technologies) for MST measurements. The thermophoresis measurements were carried out using NanoTemper Monolith NT115 (NanoTemper Technologies) with 80% LED power and 40% IR-Laser power. At least three independent experiments were performed. Initial MST data were processed using Monolith NT115, and dissociation constant (K_d_) was determined using KalidaGraph by fitting the following equation:

1 $$y=\frac{m1+(m2-m1)}{(1+\frac{m3}{x})}$$

where m1 is the thermophoresis reading of the labeled cochaperonin in the absence of the unlabeled titrating protein, m2 is the thermophoresis reading when all the labeled cochaperonin was bound with the unlabeled titrating protein, and m3 is the K_d_.

### *In vivo* complementation assay

The MGM100 *E*. *coli* cell strain (kanamycin resistant, Kan^R^) was obtained from the *E*. *coli* Genetic Stock Center at Yale University. pTrc is a *lac* promoter-based expression vector; the *lac*-based vector pBbE5c^44^ was used to express GroES, GroES^7^ and GroES^7^ variants. CaCl_2_ competent MGM100 cells were co-transformed with both plasmids and plated onto LB agar containing 50 μg/mL kanamycin, 100 μg/mL ampicillin, 50 μg/mL chloramphenicol, and 0.2% w/v arabinose. Conditions for cell growth and titration are described in ref. 29.

### Data availability statement

The datasets generated during and/or analysed during the current study are available from the corresponding author on reasonable request.

## Electronic supplementary material

Supplementary Information
