# Supplementary material for: Creating the Functional Single-Ring GroEL-GroES Chaperonin Systems via Modulating GroEL-GroES Interaction
Source: Sci Rep. 2017 Aug 29;7:9710. doi: 10.1038/s41598-017-10499-4 (PMC5575113; doi:10.1038/s41598-017-10499-4)
Supplement: Supplementary file 1 — Supplementary Information [file 41598_2017_10499_MOESM1_ESM.pdf]

Creating the Functional Single-Ring GroEL-GroES Chaperonin Systems via Modulating GroEL-GroES Interaction

Melissa Illingworth[1], Holly Ellis[2], Lingling Chen[1,3,*]

[1] Department of Molecular and Cellular Biochemistry and [2] Biology Department, Indiana University, Bloomington, IN 47405

[3] Department of Chemistry, College of Chemistry and Chemical Engineering, Xiamen University, Xiamen, China, 361005

*, Corresponding Author

Correspondence to linchen@indiana.edu

Supplementary Table S1 Biochemical characterizations of the double-ring GroEL-GroES and GroEL-GroES^7 variants.

Samples	ATPase activity (min^{-1}) and (%)	MDH yield[a] (%)	Binding affinity K_d (nM)
GroEL	0.43 ± 0.05 (100 ± 12.3)	N. A.	N. A.
GroEL + GroES	0.21 ± 0.02 (48.7 ± 4.4)	53.9	3.83 ± 0.93
GroEL + GroESI25A	0.43± 0.01 (98.1 ± 2.5)	2.37	6,190 ± 358
GroEL + GroESL27A	0.43 ± 0.04 (98.9 ± 8.1)	2.86	5,540 ± 442
GroEL	0.39 ± 0.04 (100 ± 10.5)		
GroEL + GroES	0.17 ± 0.02 (42.5 ± 5.3)		
GroEL + GroES^7	0.21 ± 0.01 (53.2 ± 2.7)	79.0	3.05 ± 0.50
GroEL + GroES^{7}I25A$_1$	0.21 ± 0.02 (53.2 ± 4.3)	71.6	4.60 ± 0.76
GroEL + GroES^{7}I25A$_{1,2}$	0.23 ± 0.02 (58.5 ± 6.2)	65.3	8.97 ± 1.6
GroEL + GroES^{7}I25A$_{1,3}$	0.23 ± 0.03 (58.5 ± 6.4)	62.0	10.0 ± 2.5
GroEL + GroES^{7}I25A$_{1,4}$	0.25 ± 0.02 (62.8 ± 5.3)	64.5	8.99 ± 1.7
GroEL + GroES^{7}I25A$_{1,4,6}$	0.31 ± 0.02 (79.0 ± 4.4)	29.5	N. D.
GroEL + GroES^{7}I25A$_{1,4,7}$	0.33 ± 0.01 (82.9 ± 1.7)	36.1	77.2 ± 21
GroEL	0.42 ± 0.05 (100 ± 11.0)	N. A.	N. A.
GroEL + GroES	0.19 ± 0.03 (46.5 ± 6.0)		
GroEL + GroES^7	0.20 ± 0.01 (48.0 ± 1.3)		
GroEL + GroES^{7}I25D$_1$	0.25 ± 0.02 (60.0 ± 5.9)	74.3	4.07 ± 0.80
GroEL + GroES^{7}I25D$_{1,2}$	0.30 ± 0.01 (72.6 ± 1.2)	N. A.	9.24 ± 1.0

GroEL + $GroES^7I25D_{1,3}$	0.31 ± 0.01 (74.2 ± 2.5)	78.9	13.3 ± 2.9
GroEL + $GroES^7I25D_{1,4}$	0.32 ± 0.04 (76.1 ± 10.6)	60.0	9.58 ± 2.0
GroEL + $GroES^7I25D_{1,4,6}$	0.34 ± 0.02 (82.3 ± 6.5)	16.4	N. D.
GroEL + $GroES^7I25D_{1,4,7}$	0.33 ± 0.03 (78.2 ± 6.9)	21.5	40.0 ± 15

[a], averaged over the last two time points (45 and 60 min)

N. A., not available

N. D., not detected

Supplementary Table S2 Biochemical characterizations of the single-ring $GroEL^{SR}$-GroES and $GroEL^{SR}$-$GroES^{7}$ variants.

Samples	ATPase activity (min^{-1}) and (%)	MDH yield[a] (%)	Binding affinity K_d (nM)
$GroEL^{SR}$	0.68 ± 0.07 (100 ± 10.1)	1.16	N. A.
$GroEL^{SR}$ + GroES	0.06 ± 0.03 (8.7 ± 3.6)	15.0	3.67 ± 2.2
$GroEL^{SR}$ + $GroES^{7}$	0.04 ± 0.03 (5.4 ± 3.7)	13.5	4.54 ± 2.3
$GroEL^{SR}$ + $GroES^{7}I25A_{1}$	0.14 ± 0.01 (20.0 ± 1.7)	20.4	8.50 ± 3.3
$GroEL^{SR}$ + $GroES^{7}I25A_{1,2}$	0.33 ± 0.01 (48.2 ± 1.9)	45.6	35.0 ± 11
$GroEL^{SR}$ + $GroES^{7}I25A_{1,3}$	0.33 ± 0.07 (49.0 ± 9.6)	30.9	39.7 ± 19
$GroEL^{SR}$ + $GroES^{7}I25A_{1,4}$	0.36 ± 0.06 (52.4 ± 8.8)	50.0	51.3 ± 26
$GroEL^{SR}$ + $GroES^{7}I25A_{1,4,6}$	0.53 ± 0.05 (77.1 ± 7.3)	7.01	N. D.
$GroEL^{SR}$ + $GroES^{7}I25A_{1,4,7}$	0.54 ± 0.02 (78.3 ± 2.6)	7.46	N. D.
$GroEL^{SR}$	0.70 ± 0.07 (100 ± 10.0)		
$GroEL^{SR}$ + GroES	0.05 ± 0.03 (7.1 ± 4.0)		
$GroEL^{SR}$ + $GroES^{7}$	0.10 ± 0.03 (14.3 ± 4.0)		
$GroEL^{SR}$ + $GroES^{7}I25D_{1}$	0.14 ± 0.03 (19.8 ± 4.1)	28.4	25.6 ± 9.8
$GroEL^{SR}$ + $GroES^{7}I25D_{1,2}$	0.45 ± 0.02 (64.0 ± 3.5)	20.8	38.7 ± 6.2
$GroEL^{SR}$ + $GroES^{7}I25D_{1,3}$	0.46 ± 0.01 (65.1 ± 2.2)	25.3	71.9 ± 22
$GroEL^{SR}$ + $GroES^{7}I25D_{1,4}$	0.43 ± 0.02 (61.0 ± 3.3)	58.0	73.1 ± 27
$GroEL^{SR}$ + $GroES^{7}I25D_{1,4,6}$	0.54 ± 0.02 (77.0 ± 2.5)	3.43	N. D.
$GroEL^{SR}$ + $GroES^{7}I25D_{1,4,7}$	0.56 ± 0.01 (79.5 ± 1.4)	9.51	N. D.

[a], averaged over the last two time points (45 and 60 min)

N. A., not available

N. D., not detected
